# Hybrid gMLP model for interaction prediction of MHC-peptide and TCR

**DOI:** 10.3389/fgene.2022.1092822

**Published:** 2023-01-04

**Authors:** Lichao Zhang, Haojin Li, Zhenjiu Zhang, Jinjin Wang, Gang Chen, Dong Chen, Wentao Shi, Gaozhi Jia, Mingjun Liu

**Affiliations:** ^1^ School of Intelligent Manufacturing and Equipment, Shenzhen Institute of Information Technology, Shenzhen, China; ^2^ School of Software, Shandong University, Jinan, China; ^3^ Tianjin Universiity, Tianjin, China

**Keywords:** gMLP, deep learning, MHC-peptide and TCR interaction, feature representation, artificial intelligence

## Abstract

Understanding the interaction of T-cell receptor (TCR) with major histocompatibility-peptide (MHC-peptide) complex is extremely important in human immunotherapy and vaccine development. However, due to the limited available data, the performance of existing models for predicting the interaction of T-cell receptors (TCR) with major histocompatibility-peptide complexes is still unsatisfactory. Deep learning models have been applied to prediction tasks in various fields and have achieved better results compared with other traditional models. In this study, we leverage the gMLP model combined with attention mechanism to predict the interaction of MHC-peptide and TCR. Experiments show that our model can predict TCR-peptide interactions accurately and can handle the problems caused by different TCR lengths. Moreover, we demonstrate that the models trained with paired CDR3β-chain and CDR3α-chain data are better than those trained with only CDR3β-chain or with CDR3α-chain data. We also demonstrate that the hybrid model has greater potential than the traditional convolutional neural network.

## Introduction

T cells are key mediators for initiating effective adaptive cell-mediated immune responses. T cells are required to continuously recognize antigens in lymphoid and peripheral tissues, such as peptides or lipids displayed by major histocompatibility complex (pMHC) molecules of other cells. The major histocompatibility complex (MHC) is to expose the protein antigen fragments inside the cell to the surface of the cell (Li et al., 2019; Liu et al., 2020). The T-cell antigen receptor binds to the MHC to detect whether the cell is normal and to determine whether to activate the host’s immune system to kill infected or malfunctioning cells (Cao et al., 2021; Zhang Q. et al., 2022). TCR mainly contains two peptide chains: *α*-chain and *β*-chain. Each peptide chain is mainly divided into the V region and C region, *etc.* The specificity of the TCR molecule is mainly found in the V region, which consists of CDR1, CDR2, and CDR3 rings. Existing experiments show that CDR1 and CDR2 always bind to MHC (Feng et al., 2007; Rossjohn et al., 2015; La Gruta et al., 2018) molecules, and CDR3 binds to antigen fragments. CDR3 has the greatest specificity and therefore becomes the decisive factor.

Many studies related to the prediction of TCR-pMHC interactions have been published (Dash et al., 2017; De Neuter et al., 2018; Jurtz et al., 2018; Gielis et al., 2019; Jokinen et al., 2019; Moris et al., 2019; Wong et al., 2019; Fischer et al., 2020; Springer et al., 2020; Tong et al., 2020; Montemurro et al., 2021). They provide a wide range of data and modeling techniques. Most were constructed based on the data from IEDB, VDJdb, and McPAS TCR. In addition to epitope information, individual CDR3β sequences (Jurtz et al., 2018; Springer et al., 2020; Tong et al., 2020), mixtures of CDR3α- and CDR3β sequences (Moris et al., 2019), or smaller datasets entailing all six CDR3 sequences and potentially additional cellular information were utilized. (Jokinen et al., 2019; Fischer et al., 2020). Glanvolle and Dash et al. (Dash et al., 2017; Lanzarotti et al., 2019) examined clear features of short amino acid motifs in the CDR3 region of TCRα- and TCRβ-chains, demonstrating the specific peptides presented by MHC molecules. Recent studies have introduced machine learning algorithms into these motifs to predict the interaction of peptide molecules with specific TCR molecules based on the motifs. These algorithms include similarity distance algorithm (TCRdist) (Dash et al., 2017), Gaussian process classification methods (TCRGP) (Jokinen et al., 2019), random forest (TCRex) (De Neuter et al., 2018; Gielis et al., 2019), k-mer feature spaces in combination with PCA and decision trees (SETE) (Tong et al., 2020). More complex machine learning methods include convolutional neural network (NetTCR, ImRex, NetTCR2.0) (Jurtz et al., 2018; Moris et al., 2019; Montemurro et al., 2021) and natural language processing (NLP) methods (ERGO) (Springer et al., 2020; Matougui et al., 2021). However, recent studies mostly use machine learning methods or simple deep learning models such as CNN and LSTM instead of Transformers (Vaswani et al., 2017), gMLP (Liu et al., 2021), and other models with better performance. There is still room for improvement in terms of the prediction performance. On the other hand, most of the publicly available TCR-pMHC specific data are located in Immune Epitope Database (IEDB) (Vita et al., 2019), McPas-TCR (Bagaev et al., 2020), VDJdb (Tickotsky et al., 2017), and other databases. However, these databases mainly contain CDR3β-chain data, lacking paired *α*- and *β*-chain data. Esteban Lanzarotti et al. (Dash et al., 2017; Lanzarotti et al., 2019) recently improved the prediction performance of the TCR target prediction model by integrating the information of paired *a*- and *ß*-chain, indicating the importance of the information about paired *a*- and *ß*-chain. To study the specificity of pMHC on paired *a*- and *ß*-chains, single cell (SC) technique is required. However, it costs much more, while much less pair-specific data is publicly available. This is a critical shortcoming of current databases, highlighting the urgent need for further development of cost-effective SC technologies to generate accurate high-quality paired data (Bentzen et al., 2016).

Although most of the major histocompatibility complex class I (MHCI) alleles have relatively reliable predictions for binding to peptides, prediction models for T-cell receptor (TCR)-peptide interactions remain poor due to the limited training data available. In this work, we constructed a novel gMLP-based model combined with attention mechanism to predict the interaction of MHC-peptide and TCR. It can be applied to individual T-cell receptor sequences as well as to the entire T-cell repertoire. Results demonstrate that the patterns learned from the data can be used to describe and predict the interactions between the T-cell receptors and peptides.

## Methods and materials

### Datasets

The data used in this work were primarily collected and processed by NetTCR2.0, including paired CDR3α-chain data and *ß*-chain data, as well as *ß*-chain only data. The *ß*-chain data is from the CDR3β sequence set with the HLA-A*02:01 allele presented on the Immune Epitope Database (IEDB) on 29 January 2020. This dataset was collected and processed by NetTCR and consists of 9204 distinct CDR3β-sequences, and each sequence is labeled as a single pMHC complex. Negative sample data are generated by creating internal error combinations of TCRs and peptides, i.e., combining TCR sequences with peptides that are different from their cognate targets. An initial negative dataset was constructed from the HLA-A*02:01-restricted peptides and filtered to only include TCR-peptide pairs with UMI counts ≤10. This data set comprised 1,325,949 distinct peptide-CDR3β pairs with 69,847 unique CDR3β sequences and 19 different peptides of which seven were shared with the IEDB peptides. Positive and negative training data points were reduced to peptide-TCR pairs with CDR3β lengths within the range of 8–18 amino acids, and peptides of length equal to nine amino acids shared between the two data sets (7 peptides). These combinations were performed by extracting a list of peptide targets from the positive dataset (repeated if peptides were found to interact with multiple TCRs), and then pairing each TCR with a randomly selected peptide from the list that is different from the cognate target.

The paired positive data of *a*-and *ß*-chains were obtained from IEDB and VDJdb databases on 26 August 2020 and 5 August 2020, respectively. There are 93,859 binding pairs from IEDB and 2,843 binding pairs from VDJdb in the length range of 8–18. VDJdb is an open and comprehensive database containing over 40,000 TCR sequences and over 200 homologous epitopes as well as specific MHC allotypes obtained by manual processing of published studies. Negative sample data from 10X identified 627,323 distinct binding sites with 0 UMI counts for all peptides. A total of 33,017 different TCRs and 19 different peptides were included, 17 of which were identical to the peptides in the positive sample data. In addition, the additional dataset generated by using MIRA analysis was provided by Klinger et al. (Lanzarotti et al., 2019). This dataset consists of 379 HLA-A*02:01 related data, covering 16 peptides and 379 TCR sequences from five donors. Among them, the negative data comes from a subset of the above negative dataset. At the same time, since 62% of IEDB data only used the allele HLA-A*02:01, and the majority of HLA-A*02:01 alleles had peptides of 9-mer length. Therefore, the dataset used all HLA-A*02:01 and 9-mer peptides.

To avoid overfitting and overestimation of model performance, the entire dataset was divided into multiple different sets before model training. TCRs with more than 90% sequence identity determined by BLASTP were stored in the same data partition. The TCRs were divided into 90% and 95% datasets based on the direct average similarity of the *a*- and *ß*-chains. The three most abundant peptides in the dataset were NLVPMVATV (NLV) from human herpesvirus 5 (cytomegalovirus), GILGFVFTL (GIL) from influenza A virus, and GLCTLVAML (GLC) from human herpesvirus 4 (Epstein-Barr virus). These three represent 99% and 92% of the *ß*-chain and paired-chain data, respectively.

### Model architecture

Neural network model has powerful performance in extracting features from data and predicting data (Lv et al., 2021; Zhang Z. Y. et al., 2022). Its main advantages is the ability to optimize functions in the network by learning sequence data so that it can learn the mapping capabilities required by the task to complete the classification task. In addition, it also has strong generalization ability, and also has good prediction ability for untouched and noisy samples (Wang et al., 2021; Zulfiqar et al., 2021). At the same time, the non-linear mapping ability of neural networks can greatly simplify the difficulty of model design.

Some of the latest deep learning models such as Transformers, gMLP, and other models have achieved fairly good results in picture classification, masked language modeling (MLM), NLP, and other tasks (Dao et al., 2021; Jin et al., 2022; Lv et al., 2022; Wang et al., 2022; Wei et al., 2022). Therefore, in this paper, we hope to introduce the latest gMLP model with attention mechanism into the prediction of TCR-peptide interaction to accurately identify their interaction targets, extract the similarity between them, and improve the prediction performance. Our experiments prove that our model has more advantages than relatively simple traditional deep learning models such as Convolutional Neural Network (CNN), Long Short Term Memory (LSTM), *etc.* The area under the ROC curve (AUC) is improved by 0.07.

**FIGURE 1:**
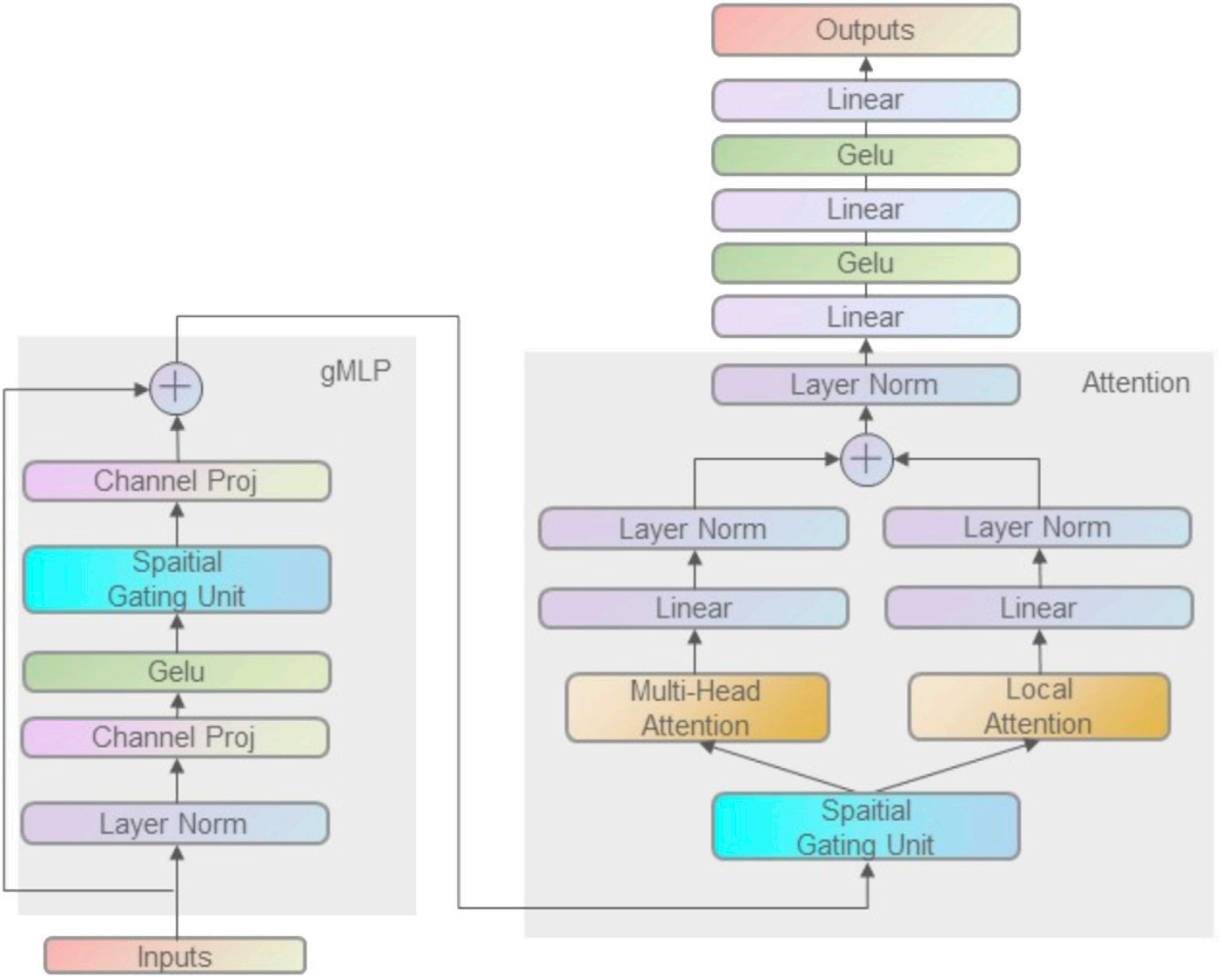
gMLP model framework with attention mechanism. The TCR CDR3 sequences and peptide sequences were encoded by BLOSUM50 matrix as model input. Firstly, the spatial information was obtained by gMLP, and then Multi-head Attention and Local-Attention were used to extract the correlation information of TCR CDR3 and peptide sequences. Finally, the output of is obtained by interleaving fully connected layers and Gelu layers.

Compared with the usual fully connected neural network, the structure of convolutional neural networks is characterized by the convolution layer and pooling layer. The biggest difference between the convolutional layer and the fully connected layer is that the neurons in the convolutional layer are not connected to all the neurons in the next layer, but pass information to the next layer through a special structural feature map. The feature map is a matrix composed of multiple neurons that share a set of weights between neurons, which is the convolution kernel of the convolutional layer. Meanwhile, the convolutional layer can contain several feature maps. In the initialization of convolutional neural networks, the weights of convolutional kernels are generally initialized by random seeds. The advantage of this approach is that it can reduce the connections between layers and prevent the network from overfitting. Pooling layer also plays an important role in convolutional neural networks. It includes both average pooling and maximum pooling. For example, the maximum pooling layer prevents the model from overfitting by selecting the parameters with the maximum information in a certain range. The specific operation process of the convolution layer is as follows: the convolution kernel slides to all positions on the image, and does an inner product with the pixel and its domain pixels at each position. Convolution is widely used in image processing. Different convolution kernels can extract different features, such as edge, linearity, angle, *etc.* In deep convolutional neural networks, low-level to complex features of images can be extracted by convolution operations. The framework of the model is illustrated in Figure 1.

#### Feature encoding for TCR and peptide

BLOSUM50 matrix (Henikoff and Henikoff, 1992) was used to encode *a*-chain, *ß*-chain, and peptide sequences into matrices. That is, each amino acid is represented as the score for substituting the amino acid with all the 20 amino acids. The BLOSUM encoding scheme maps a sequence of length l into an array of dimension l×20 . BLOSUM: First, find the amino acid pattern, i.e., a meaningful amino acid fragment (e.g. a domain and its two adjacent amino acid sequence fragments). Amino acid conservation (data on the substitution of one amino acid for another) was compared between identical amino acid patterns, and then BLOSUM50 matrix was generated based on the comparison data between all 50% conserved amino acid patterns.

#### gMLP

Transformers have made many breakthroughs in natural language processing and have been shown to work well in computer vision. Due to this success, Transformer has largely replaced LSTM-RNN as the default architecture for NLP. The gMLP is one of the most important architectural innovations in deep learning. They questioned the necessity of the existing Transformer attention layer and proposed an attention-free network architecture gMLP with spatially gating units, which achieved comparable performance to Transformer on both image classification and masked language modeling tasks. And if a little bit of attention is added to gMLP—a single-head attention with size up to 128 is sufficient to make gMLP outperform Transformers on all NLP tasks. Meanwhile, the future of gMLP is promising because the spatial filters of gMLP are simpler and achieve better performance with fewer network parameters than Transformer’s black-box matrix.

gMLP is composed of many blocks with identical size and structure. Here we use three identical blocks. The *a*-chain, *ß*-chain and peptide sequences encoded as matrices are input into the three blocks to extract the features. Each block is expressed as follows:

$$\begin{array}{l} Z=\sigma \left(XU\right) \end{array}$$ (1)

$$\begin{array}{l} \tilde{Z}=s\left(Z\right) \end{array}$$ (2)

$$\begin{array}{l} Y=\tilde{Z}V \end{array}$$ (3)

where σ is an activation function such as GeLU. U and V define the linear projections along the channel dimension is the same as the FFN of Transformer. s is the Spatial Gating Unit. When s is an identity mapping, the above transformation degenerates to a regular FFN, where individual tokens are processed independently without any cross-token communication. Therefore, the spatial gating unit is mainly designed with a good capability to capture complex spatial interactions across tokens. Each block first normalizes the input matrix of *a*-chains, *ß*-chains and peptide sequences using LayerNorm, normalizing the dimension to stabilize the layer (The reason for not using BatchNorm is that the normalized statistics of LayerNorm are independent of the batch size when the batch is small, and the resulting model is more stable and regularized). After that, through a layer of channel mapping layer (the simplest fully connected layer is used here), after passing through the GeLU activation function, the data is sent to the most critical Spatial Gating Unit (SGU) in the block to capture the spatial interactions. Then a channel mapping layer is added to the original input to obtain the feature output in the sequence. The output is obtained after the feature extraction of three identical blocks. Among them, the SGU uses a simple spatial linear mapping containing operations on spatial dimensions, enabling it to perform cross-token operations. The spatial gating unit first divides the input data into two parts from the channel dimension. One part is linearly mapped to the space, and then the output is obtained by dot product with the other part. For training stability, in the initialization parameters, we uniformly sampled W in the range of [−0.01,0.01] (gMLP was initialized around 0), and b was initialized to 1. This enables each block to behave the same as a regular fully connected layer in the early stage of model training, so that each token is processed separately, and only cross-token spatial information is gradually added in the training process.

The overall formulation of spatial gating unit (SGU) is similar to Gated Linear Units (GLUs), as well as earlier works including LSTM and RNN. A key difference is that gating is computed based on the projection over the spatial dimension rather than the channel (hidden) dimension. SGU is also related to the Squeeze-and-Excite (SE) block in terms of element-wise multiplication. However, unlike SE blocks, SGU does not contain cross-channel projections at all, nor does it enforce permutation invariance (a key feature of the content-based attention module) because it statically parameterizes the spatial transformation. In theory, spatial projections in SGU can learn special depthwise convolutions. Unlike typical depthwise convolutions that include channel filters, SGU only learns a single transformation shared across channels. Finally, we note that SGU provides an alternative mechanism to capture higher-order relations in addition to self-attention. Specifically, the output of the equation contains up to 2nd-order interactions, while the output of self-attention (assuming no non-linearity) contains up to third-order interactions. In terms of computational cost, SGU has $n^{2}e/2$ multiplication and addition, which is comparable to the $2n^{2}d$ of dot-product self-attention. The two are linear over the input channel size.

#### Local attention

To extract the local potential feature information in the sequence, we introduce the local attention mechanism (Luong et al., 2015; Sharma and Srivastava, 2021; Chen et al., 2022). Local-Attention only pays attention to some hidden states and does not calculate attention scores for all information. Instead, it selectively focuses on a custom context window, and only calculates the attention score for the information in the window, which can reduce the computational cost. The window size used in this paper is 2. First, we used the peptide sequence processed by gMLP as the Query of Local-Attention after a spatial mapping layer and normalization using LayerNorm. After that, we use a window of size two to loop and take the *a*-chains and *ß*-chains splicing data processed by gMLP as the Key and Value of Local-Attention. For the Key and Value from each window along with the previous Query , we use the same method as Multi-head Attention to get the attention information. Finally, the information obtained from all windows was superimposed. The final attention information was obtained by passing a spatial mapping layer again and using LayerNorm normalization to prevent overfitting of the model.

## Results

### Model performance on CDR3β data

Our model identifies one or more specific peptides interacting with TCRs from a large uncorrelated TCR dataset. We selected three of the most common IEDB peptides (GILGFVFTL, GLCTLVAML and NLVPMVATV) and tested the possibility of randomly selected TCR-peptide interactions. The peptides for these test models were obtained from the MIRA dataset.

We trained our model using data containing only the TCR CDR3β-chains in our initial model evaluation. One of the key points to model training and evaluation is the quality of the data. Therefore, we used the same method as NetTCR2.0 to train and test the model on different CDR3β datasets by cross-validation. The datasets were also divided into different thresholds based on the similarity between the test set and the training set. In addition, we also used external datasets for performance evaluation.

**FIGURE 2:**
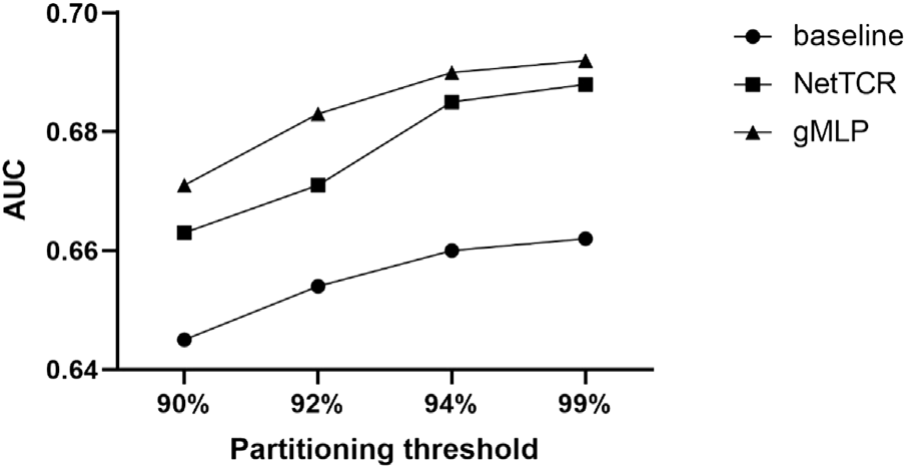
Prediction performance of NetTCR, baseline, and gMLP models are compared using AUC in the IEDB dataset with four different thresholds of 90%, 92%, 94%, and 99% similarity, respectively.

We compared three models. One is our deep learning model, gMLP model with attention mechanism. The other one is sequence similarity baseline model (a simple similarity-based method model with kernel score, in which the predicted score for a given TCR was calculated as the highest score obtained when scoring CDR3β against a positive CDR3β database. In 5-fold cross-validation, each of the five partitions in turn represented a test set, and the positive elements in the remaining four partitions defined the database. For external evaluation, all positive elements in the training dataset defined the database. For analysis of paired *a* and *ß* TCR sequences, the similarity scores were calculated as the highest average of individual *a* and *ß* CDR3 sequence scores for each TCR), The last one is the CNN-based NetTCR model. We compared these three models to show the strong advantages of the gMLP model with attention mechanism over the simple models (see Figure 2). Experiments showed that our model significantly outperformed the baseline model and the NetTCR model at different thresholds of 90%, 92%, 94%, 99%, and the AUC was 0.04 higher, which illustrated the significant advantage and powerful performance of complex deep learning models for existing classification problems. At the same time, with the increase of the threshold, the similarity between the training set and test set increased, and the performance of the three models also improved.

**FIGURE 3:**
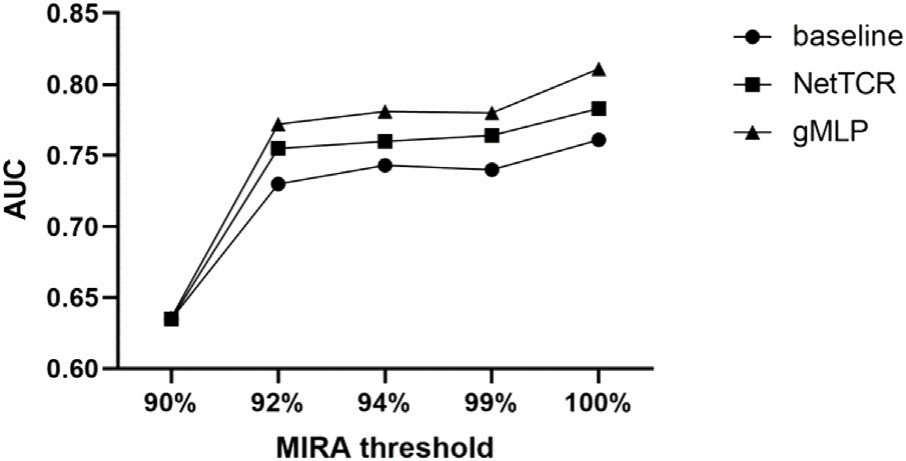
Comparing the prediction performance of baseline, NetTCR, and gMLP models on different thresholds of similarity between the training and test sets in the MIRA dataset.

Afterwards, we evaluated the three models on different thresholds of 90%, 92%, 94%, 99%, and 100% for the MIRA dataset (see Figure 3). The results showed that the performance of the models tested in the MIRA dataset was improved compared to the IEDB dataset with different thresholds, and the AUC had increased by 0.1, which indicated the better quality of the data in the MIRA dataset. Similarly, the performance of the model improved as the threshold increases.

**FIGURE 4:**
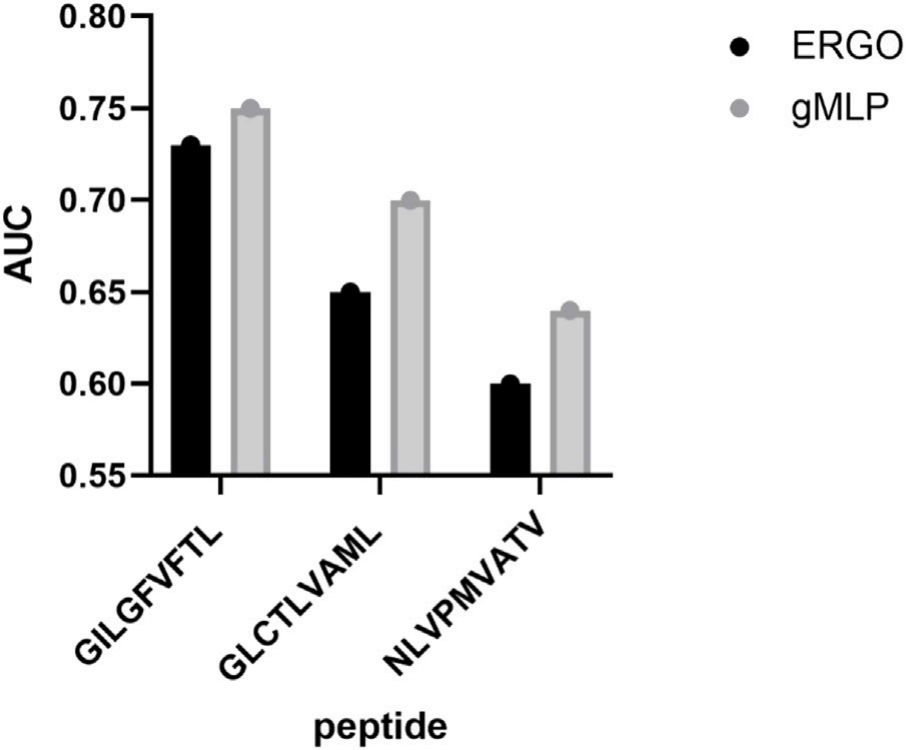
Comparison of NetTCR and ERGO-LSTM prediction performance. The AUC of the model on the three most common peptides in the IEDB dataset was compared.

To further compare other simple deep learning models, we compared our model with ERGO deep learning model, which is more complex than CNN. The ERGO model used a more complex Long Short Term Memory network (LSTM) or AutoEncoder (AE) model for TCR-peptide interaction prediction on the VDJdb dataset. We trained ERGO using LSTM and gMLP with attention mechanism on the IEDB dataset, respectively. We then compared the prediction performance of these two models on three different peptides (see Figure 4). The overall AUC of gMLP model with attention mechanism was 0.71, which had a huge advantage over the ERGO model of 0.66. We then further tested all versions of ERGO (including ERGO McPAS LSTM, ERGO McPAS AE, ERGO VDJdb LSTM, ERGO VDJdb AE) on the MIRA dataset. The AUC of the gMLP model was 0.85, while the overall AUC of the ERGO was 0.7. The complexity of gMLP deep learning model is effective in improving the prediction of TCR-peptide interactions.

### Model performance on paired CDR3α and CDR3β data

**FIGURE 5:**
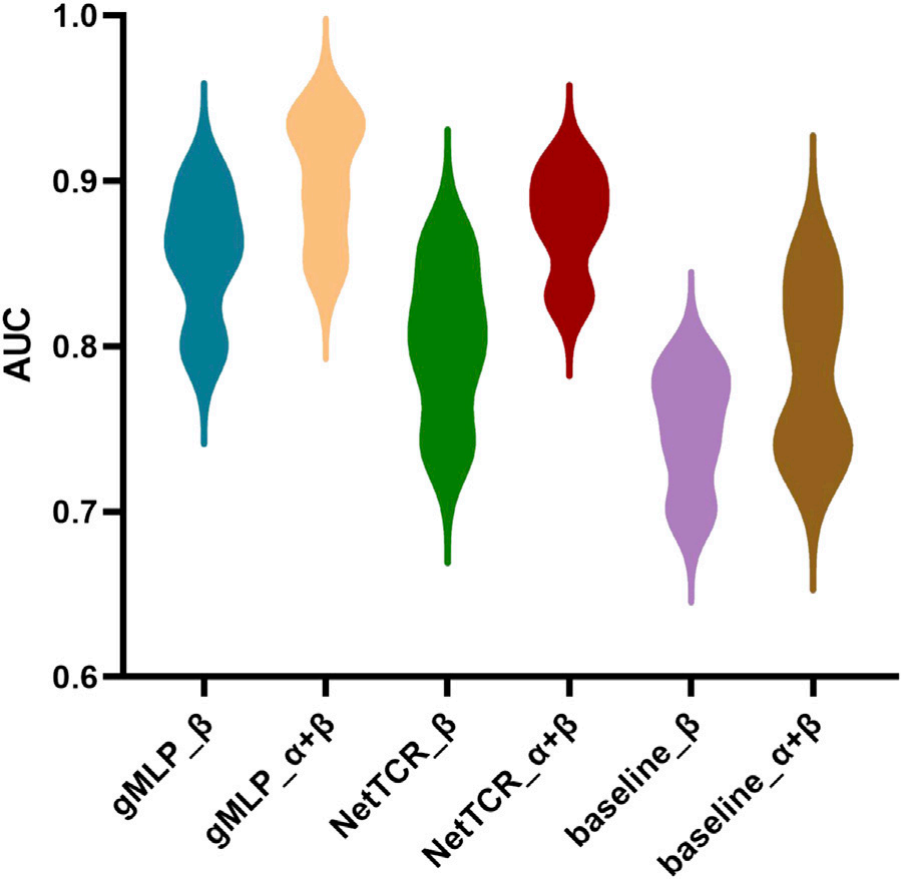
Comparison of the overall AUC obtained from gMLP, baseline, and NetTCR on data containing only *ß*-chains and data containing paired *a*-and *ß*-chains, respectively.

Compared with the dataset containing only *ß* chains, the dataset using paired *a*-chains and *ß*-chains contains more information. We used the data in NetTCR to divide the dataset according to the average similarity between *a*- and *ß*-chains, which were the datasets with 90% and 95% threshold, respectively. We compared NetTCR, baseline, and gMLP model with attention mechanism on these two datasets (see Figure 5). The prediction results of three different peptides were also compared. The experimental results show that using paired *a*-chains and *ß*-chains datasets can significantly improve the prediction performance of the model. The AUC of the three models was increased by 0.04, 0.07, and 0.06, respectively, which illustrated the importance of the information provided by paired data.

Meanwhile, the gMLP model with attention mechanism had an AUC of 0.93, which was better than NetTCR with AUC of 0.89 and baseline with AUC of 0.85. The gMLP model also significantly outperformed the other two models on the single-chain *ß* dataset. We also observed that the performance of the model decreases when the threshold was reduced from 95% to 90%. However, NetTCR was more stable on both datasets, and the AUC decreased less when the threshold was reduced. In the interaction comparison of TCR with the three most abundant peptides in IEDB, we found that the NetTCR did not perform well in the prediction of relatively small number of NLV peptides. However, the AUC of gMLP for the prediction of NLV peptides was still 0.8 or even 0.91, indicating the robustness of our model in Few-Shot learning.

In general, the gMLP model with attention mechanism we developed has good performance in predicting TCR-peptide interactions, and the low performance of several current models is due to the low quality of the CDR3β data.

**FIGURE 6:**
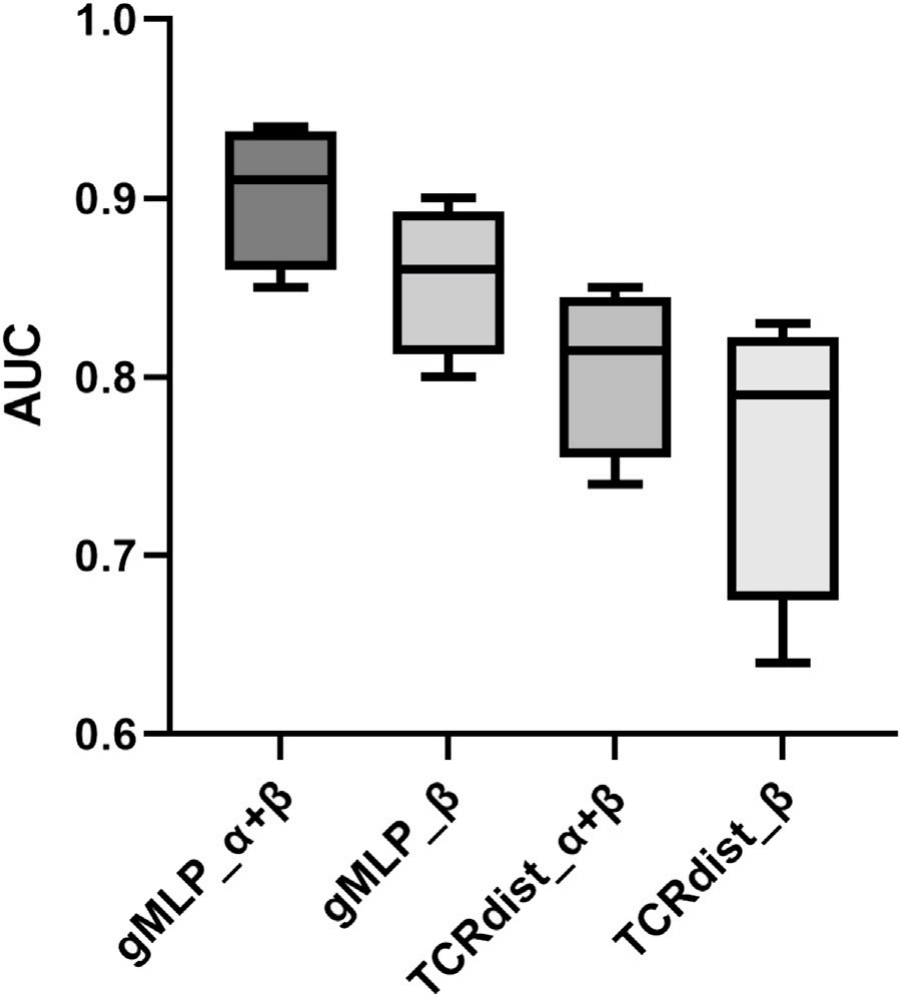
Performance evaluation of gMLP and TCRdist on the IEDB 95% threshold dataset.

Then we compared the performance of gMLP model with attention mechanism and the TCRdist model. The configuration of the TCRdist model here is the same as the baseline model, only utilizing the distance metric proposed in the TCRdist publication. We compare them on the 95% threshold dataset. The results showed that the performance of gMLP model far exceeded that of TCRdist model (see Figure 6). In the comparison of the three peptides, we found that TCRdist and NetTCR were similar in predicting the specificity of NLV peptides with a significant performance degradation compared to the other two peptides. However, the gMLP model still had good performance, indicating that the gMLP model can still learn key features in the data when there were few samples in the data set.

The model’s capability to determine the proper peptide target for a particular TCR was next tested using paired predictions of TCRs bound to GIL, NLV, or GLC with the other two peptides. For each TCR that was positive to one of the three peptides in this case, binding to GIL, NLV, and GLC was predicted (using cross-validation). From the peptide with the lowest rank value, the anticipated target for each TCR was found. The performance for each peptide was represented as the percentage of properly recognized targets for the three models trained on the CDR3 and CDR3, CDR3 alone, and CDR3 alone in this investigation. All models here outperformed chance with a correct target percentage >33%. Additionally, for all three peptides, the model that was trained on CDR3 and CDR3 performed much better than either of the other models. Meanwhile, The optimal single-chain model selection was peptide-dependent, with gMLP_a outperforming gMLP_b for the NLV peptide, in line with the findings of other studies.

## Conclusion and discussions

Identifying cognate targets of TCRs is a critical step in immune detection and cancer therapy. In this paper, we proposed a deep learning-based gMLP model with multiple Attention mechanisms to solve this problem. We predicted TCR-pMHC interactions using the amino acid sequences of the peptide and CDR3 region of the TCR chains. The performance of multiple models (such as CNN, LSTM, *etc.*) was tested using independent datasets. The test results showed that the gMLP model with attention mechanism significantly outperformed the NetTCR, baseline, and ERGO models.

**FIGURE 7:**
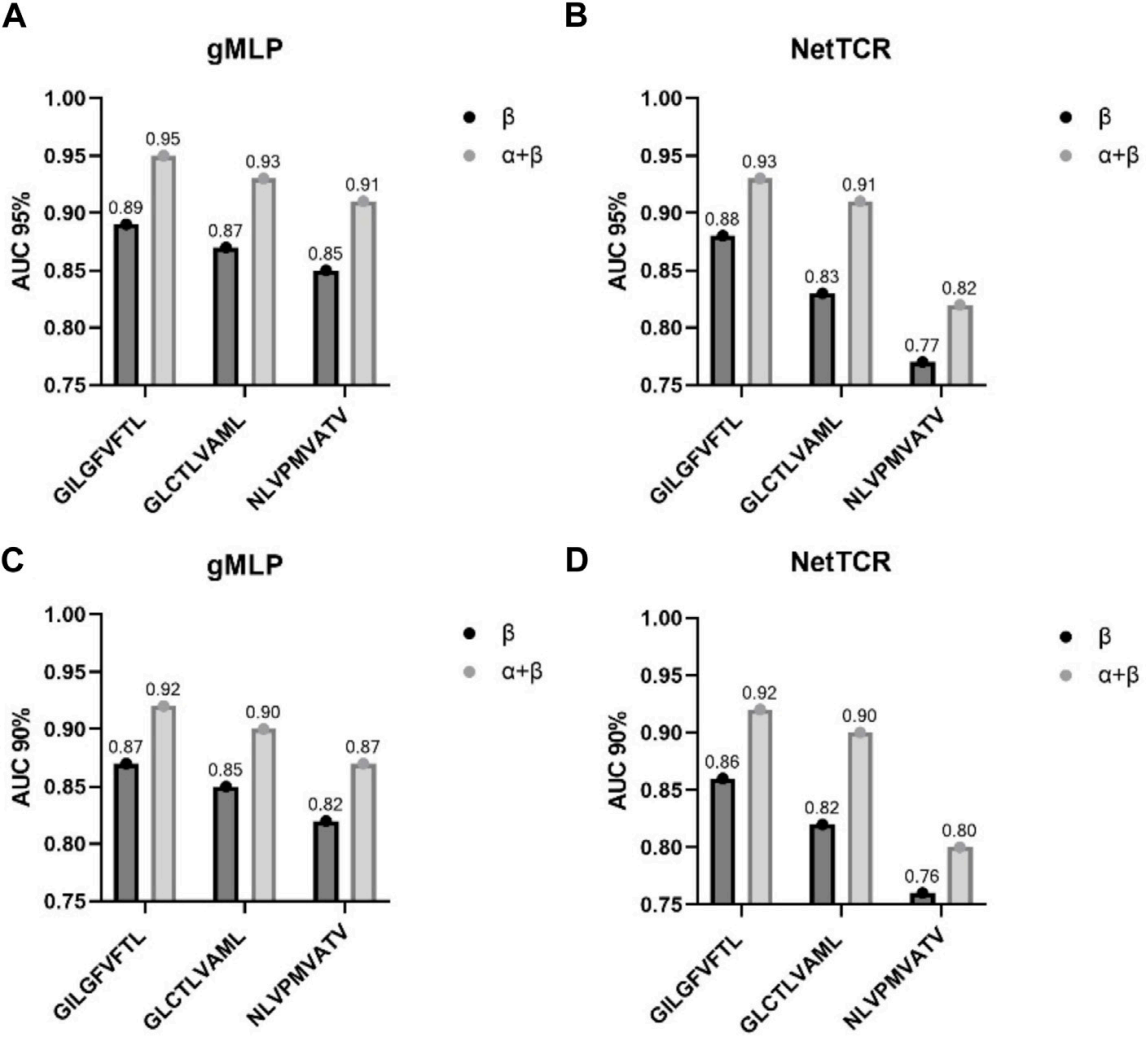
AUC from the three most common peptides in IEDB on 90%, 95% partitioned data. **(A,B)** represent the performance of gMLP and NetTCR on the 90% data. **(C,D)** represent the performance of gMLP and NetTCR on the 95% data. The partition threshold is based on the similarity between the training and test sets.

At the same time, our experiments showed that the quality of the dataset is crucial in model training. The paired CDR3α-chain and *ß*-chain data contain more important information than only *ß*-chain and *a*-chain data, which can effectively improve the performance of the model. We trained the models on the paired *a*-and *ß*-chain datasets and the dataset containing only *a*-and *ß*-chains, respectively. The experimental results show that the models trained on the paired *a*-and *ß*-chain datasets outperform the models trained on the dataset containing only single chains, reflecting that the paired *a*-and *ß*-chain datasets can provide more effective information. Additionally, in agreement with earlier research (Jurtz et al., 2018; Moris et al., 2019), the results of the current study unequivocally affirm the notion that both TCR chains contribute to TCR specificity (importantly, their relative importance is pMHC-specific), and that accurate TCR specificity prediction can only be attained by incorporating this combined information. Accordingly, we investigated the TCR-specific performance of the model and found a high level of predictive power, with an accuracy of approximately 90% for three peptides (GILGFVFTL, GLCTLVAML, and NLVPMVATV) (see Figure 7). This suggests that the model may help to address not only which TCRs are specific for a particular peptide, but also has the potential to answer which peptides are specific for a particular TCR, which has important applications in biomedical T-cell therapy (Yee, 2005; Jones et al., 2021).

Compared with relatively simple deep learning models such as NetTCR (CNN) and ERGO (LSTM), our model can not only map TCR sequences to a high-dimensional abstract feature space suitable for specific classification, but also the SGU in gMLP can capture the spatial interaction information of the data and the Attention mechanism can capture the potential correlation between TCR sequences and peptide sequences. Meanwhile, the comparison experiments with ERGO and NetTCR models showed that complex deep learning models were very necessary for classification problems. Our gMLP model can achieve the same or even higher predictive performance with fewer parameters than models such as Transformers.

The high performance of our developed gMLP model with attention mechanism has been demonstrated on pairwise TCR datasets. Due to the small number of training peptides, the model can currently only be applied to a limited set of peptides contained in the training data. Our model was tested on a dataset containing three HLA-A*02:01 peptides and achieved a prediction accuracy value of about 75%, greatly surpassing the performance of both the baseline and ERGO and NetTCR models. This result confirmed that the development of accurate prediction models for TCR specificity is contingent on the availability of paired *a*-and *ß*-sequence data and suggests that a predictive power can be achieved to the extent of practical biomedical applications. Moreover, as more data become available, we expect the predictive power of the model to improve, enabling accurate prediction of unidentified peptides as in the previous peptide-MHC interaction prediction model (Hoof et al., 2009). Finally, the presented model framework is highly flexible and allows for the straightforward integration of the MHC molecule or TCRα chain in the future when data become available, to train a truly global prediction method. Moreover, we provide a new way for application of the proposed deep learning arthitecture to address other similar problems, such as protein-protein interaction prediction (Wei et al., 2017), protein-peptide binding prediction (Wang et al., 2022), and others (Su et al., 2019a; Su et al., 2019b; Manavalan et al., 2019; Manayalan et al., 2019; Wei et al., 2020), *etc.*

## Data Availability

The original contributions presented in the study are included in the article/supplementary material, further inquiries can be directed to the corresponding authors.
